# Mechanism of High-Temperature Thickening Regulation in Amide-Modified Ultra-High-Temperature Polycarboxylate Retarders

**DOI:** 10.3390/ma19040657

**Published:** 2026-02-09

**Authors:** Youzhi Zheng, Zhanwu Zhang, Wenzhe Li, Quan Cao, Tianan Deng, Jun Zhao, Yalan Wang, Chao Mei, Rongyao Chen, Mai Xu, Miaomiao Hu, Kunliang Xie

**Affiliations:** 1 Engineering Technology Research Institute, PetroChina Southwest Oil & Gas Field Company, Guanghan 618300, China; zyz08@petrochina.com.cn (Y.Z.);; 2 School of Chemical Engineering and Technology, Tianjin University, Tianjin 300354, China; 3 Zhejiang Shaoxing Institute, Tianjin University, Shaoxing 312300, China; 4 Haihe Laboratory of Sustainable Chemical Transformations, Tianjin 300051, China

**Keywords:** deep well cementing, oil well cement, high temperature, polymer retarder, mechanism

## Abstract

**Highlights:**

**What are the main findings?**

- The high-temperature retarding effect of retarders was improved by introducing DMAA monomers.
- The chelation between copolymers and Ca^2+^ inhibits early hydration and delays hydration time.
- The stretching of polymer chains at high temperatures enhances their high-temperature performance.
- The hydrolysis of amide groups in DMAA produces carboxyl groups enhancing the retarding ability.

**What are the implications of the main findings?**

- A high-temperature resistant retarder has been developed.
- An explanation for the dynamic regulation mechanism of high-temperature retarders at high temperatures has been provided.
- Technical support for deep high-temperature cementing operations was provided.

**Abstract:**

As oil and gas well development moves towards ultra deep formations, the high temperature at the bottom of the well causes the failure of copolymer retarders, leading to increased risk of oil and gas leakage and carbon emissions during cementing operations. To further ensure the safety of high-temperature oil and gas cementing operations, the influence of N,N-dimethylacrylamide (DMAA) on the high-temperature performance of copolymer retarders was explored. DMAA was introduced into copolymer retarders to form ultra-high temperature retarders. By analyzing the micro mechanism of copolymer retarders, the regulation of high-temperature retarders on the micro hydration process of cement slurry at high temperatures was revealed. Results showed that the cement slurry containing 3.0% SH5L (Pentameric copolymer retarder-introduced DMAA) exhibits a significantly similar thickening time with 3.4% SH4L (Quaternary copolymer-retarder) at 180 °C, demonstrating superior retardation performance at a lower dosage. The ultra-high-temperature polycarboxylate retarder SH5L was prepared by introducing the DMAA, enhancing its temperature resistance and retardation performance at high temperatures. The coupling of SH5L and Ca^2+^ retards the hydration and crystallization process of the cement slurry. The combination of rigid polycyclic structures and cationic monomers weakens the chelation between anionic groups and Ca^2+^, inhibiting the curling of polymers in ionic solutions. Polymer chains stretch with increasing temperature, enhancing their ability to bind with Ca^2+^ and improving their high-temperature retardation performance.

## 1. Introduction

Oil and gas well cementing operations, as a core engineering technology in the full lifecycle of oil and gas resource exploration and development, are pivotal for ensuring reservoir sealing integrity and maintaining long-term wellbore stability [1,2,3,4]. The quality of cementing directly impacts the efficient development of oil and gas fields, safe production, and the achievement of carbon emission reduction goals. Against the backdrop where deep and ultra-deep oil and gas reservoirs have become the main focus of current exploration and development, downhole temperatures generally exceed 150 °C, with some extreme working conditions reaching over 200 °C [5,6,7,8]. This imposes unprecedented stringent requirements on the performance of cement slurry systems. The thickening time of cement slurry must precisely match the drilling operation cycle: premature thickening can lead to displacement difficulties and uneven cement sheath bonding, while excessive delay may cause slurry loss or formation fluid channeling during the waiting-on-cement period [9,10,11]. In ultra-high-temperature environments, traditional retarders often exhibit drastic attenuation or even complete failure of retardation performance due to insufficient thermal stability of their molecular structures. This phenomenon not only directly leads to uncontrolled hydration processes of cement slurry but also triggers key issues such as abnormal early strength development and insufficient late-stage bonding strength of cement stone, rendering the cement sheath unable to form a continuous and dense sealing barrier and significantly increasing the risk of interlayer isolation failure [12]. Therefore, developing novel retarders adapted to ultra-high-temperature environments and elucidating their action mechanisms under extreme conditions have become critical technical bottlenecks that urgently need to be addressed in the field of oil and gas well engineering [13,14].

Traditional retarders mainly include lignosulfonates, hydroxycarboxylic acids and their salts, sugar derivatives, etc., whose retardation mechanisms are primarily based on adsorption–complexation or nucleation inhibition effects [15]. However, when temperatures exceed 150 °C, the molecular chains of these retarders are prone to cleavage, oxidation, or conformational changes, leading to a sharp reduction in the number of effective functional groups and decreased complexation capacity, thereby significantly reducing retardation efficiency [16,17]. Studies have shown that at 180 °C, the retardation efficiency of conventional lignosulfonates decreases by over 60% compared to room temperature, and decomposition products of hydroxycarboxylic acids may even accelerate cement hydration, shortening the thickening time instead [18,19]. Such poor adaptability makes traditional retarders inadequate for ultra-deep oil and gas well cementing needs, severely restricting the development of ultra-deep oil and gas reservoirs in regions. However, the chelation ability of carboxyl groups in their molecular structures is easily inhibited in high-temperature, high-calcium ion environments, and polymer chains tend to curl, reducing active sites, thus requiring further optimization to enhance ultra-high-temperature tolerance [20,21]. To address these issues, this study focuses on the molecular design and mechanism analysis of anti-high-temperature polycarboxylate retarders, aiming to break through the poor high-temperature retarding effect limitations of traditional retarders through structural modification.

The core of polycarboxylate retarders lies in achieving retardation via specific interactions between side-chain functional groups and cement hydration products, and ultra-high-temperature environments impose higher demands on their molecular thermal stability, functional group activity, and spatial conformation regulation capabilities [22]. The introduction of amide groups in DMAA was studied to further improve the retarding ability of high-temperature retarders at high temperatures, and the effect of introducing amide groups on the high-temperature performance of retarders was explored. At the same time, based on the influence of retarders on the micro hydration of cement slurry, the retarding mechanism of high-temperature retarders with DMAA on cement slurry was explored. The microstructure evolution of cement stone was observed using scanning electron microscopy, the phase composition evolution of cement stone was tested using XRD, the hydration product content changes in cement stone were tested using TG, and the pore structure evolution of cement stone was tested using BET. Meanwhile, the conformational transition of the copolymer was simulated based on molecular dynamics simulations. The results of this study can effectively resolve the problem of cement slurry thickening out-of-control in ultra-deep oil and gas well cementing.

## 2. Materials and Methods

### 2.1. Materials

The raw materials employed in this study comprised several key components. Monomers included 2-acrylamido-2-methylApropanesulfonic acid (AMPS), itaconic acid (IA), N,N-dimethylacrylamide (DMAA), N-vinylpyrrolidone (NVP), and methacryloyl propyl trimethylammonium chloride (MAPTAC), with ammonium persulfate (APS) serving as the initiator. For pH adjustment, analytical-grade sodium hydroxide (NaOH), procured from China National Pharmaceutical Group Chemical Reagent Co., Ltd., Shanghai, China, was utilized. Jiahua G-grade oil well cement was selected as the primary cementitious material. Additionally, other additives—including quartz sand, dispersant DRS-1S, fluid loss additive DRF-2L, and suspension stabilizer DRK-410S—were supplied by the Solid Well Completion Institute of China Petroleum Engineering Technology Research Institute Co., Ltd., Beijing, China. The detailed composition of the G-grade cement is listed in Table 1.

### 2.2. Methods

#### 2.2.1. Synthesis of Polymer Retarder

**Figure 1:**
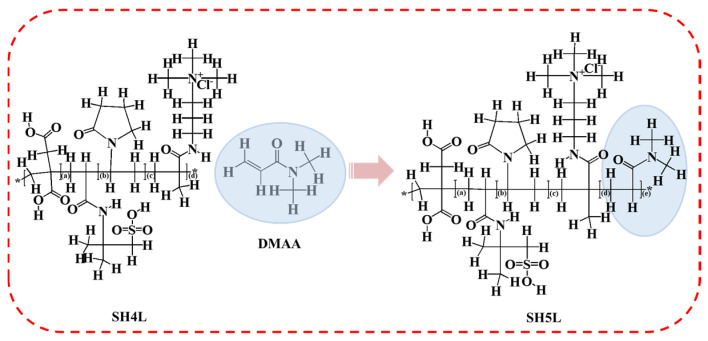
Introduced DMAA to enhance the retard performance of SH4L and form the SH5L (* is bonding site; a, b, c, d, and e respectively represent the number of repeating units).

The synthesis of the five-component high-temperature-resistant polymeric retarder SH5L proceeded as follows: first, 5.8 mol 2-acrylamido-2-methylpropanesulfonic acid (AMPS) and 2 mol itaconic acid (IA) were weighed in a predetermined ratio and dissolved in a specified volume of deionized water. The resulting mixture was preheated to 60 °C with continuous stirring, followed by the addition of sodium hydroxide (NaOH) to adjust the pH to 5–6. Subsequently, 0.5 mol N,N-dimethylacrylamide (DMAA), 1 mol N-vinylpyrrolidone (NVP), and 0.5 mol methacryloyl propyl trimethylammonium chloride (MAPTAC) were weighed in their respective proportions and added to the aforementioned solution under continuous agitation. Using ammonium persulfate (APS) as the initiator, the reaction system was kept at 80 °C for 2 h to complete polymerization, yielding the target five-component retarder SH5L. In parallel, a binary polymeric retarder (SH4L) was prepared as a control: AMPS and IA were mixed in the same ratio as SH5L, but excluding the functional monomer DMAA, with all other synthetic steps identical to those described above. The structure of SH4Land SH5L is shown in Figure 1.

#### 2.2.2. Preparation of Cement Slurry

The cement slurry was formulated in strict accordance with the guidelines of API RP 10B-2 [23] and the Chinese standard Test Methods for Oil Well Cement (GB/T 19139-2012) [24]. A commercial OWC-2000D constant-speed mixer (Manufactured by Shenyang Taige Petroleum Instrument Equipment Manufacturing Co., Ltd., Shenyang, China) was employed for the preparation procedure. Initially, the weighed cement material was homogenized, while additives were incorporated into the mixing water. Subsequently, the cement or cement/solid external additive mixture was uniformly introduced into the mixing water within 15 s at a low rotational speed of 4000 rpm, followed by high-speed stirring at 12,000 rpm for 35 s to obtain the final cement slurry.

#### 2.2.3. Characterization of Structure

Fourier transform infrared (FTIR) spectroscopy, thermogravimetric analysis (TGA), X-ray diffraction (XRD), scanning electron microscopy (SEM), and nitrogen adsorption–desorption analysis were employed to characterize the synthesized retarder and its effects on cement properties, with experimental procedures optimized for academic rigor as follows.

For FTIR analysis, the retarder sample was first dialyzed against a membrane with a molecular weight cut-off of 3500 Da for 3 days to remove small-molecule impurities. Subsequent steps included lyophilization (freeze-drying) and grinding into a fine solid powder. The polymer powder was then mixed with potassium bromide (KBr) powder at an appropriate mass ratio and compressed into transparent pellets. Spectral acquisition was performed using a NEXUS FT Fourier Transform Infrared Spectrometer (Thermo Fisher Scientific, Waltham, MA, USA), recording absorption spectra over the wavenumber range of 400–4000 cm^−1^ with 32 scans.

TDA305 gel permeation chromatograph (GPC) (Malvern Instruments Ltd.) from the Malvern of United Kingdom was used to test molecular weight, equipped with a differential detector. Polyethylene glycol with different molecular weights was used as the standard to generate a third-order standard curve. The retarder sample was dissolved in a 0.1 mol/L sodium nitrate solution as the mobile phase, with a sample flow rate of 0.5 mL/min.

Thermal stability of the polymer was evaluated via TGA using a TG209F3 instrument (NETZSCH Instruments GmbH, Selb, Germany). Testing conditions were set as follows: heating rate of 10 °C/min, nitrogen atmosphere (flow rate: 10 mL/min), and temperature range of 25–800 °C.

Phase composition of the solidified cement sample was analyzed by XRD. The sample was ground into powder and examined using a Rigaku SmartLab SE X-ray diffractometer (Rigaku Corporation, Tokyo, Japan) with Cu Kα radiation (λ = 1.5406 Å). Scans were conducted over a 2 θ range of 5–70° under a tube voltage of 40 kV and current of 30 mA.

Microstructural characterization of the cured cement slurry was performed via SEM. The sample was crushed, and a smooth central section (avoiding surface irregularities) was selected and observed using a SU8100 field-emission scanning electron microscope (Hitachi, Tokyo, Japan) at appropriate accelerating voltages.

Specific surface area and pore size distribution were determined by nitrogen isothermal adsorption–desorption analysis. The dried cement sample was ground and sieved through a 200-mesh sieve to obtain uniform powder. A known mass of powder was degassed at 80 °C for 6 h to remove adsorbed moisture, then tested using a TriStar 3000 fully automatic surface area and porosity analyzer (Micromeritics, Norcross, GA, USA). Pore size and distribution were calculated via the Barrett–Joyner–Halenda (BJH) model based on the adsorption branch of the isotherm.

#### 2.2.4. Testing of Cement Slurry Performance

The assembled slurry cup was placed in a high-temperature and high-pressure thickening instrument (model TG-8040B, manufactured by Shenyang Taige Petroleum Instrument Equipment Manufacturing Co., Ltd., Shenyang, China), and the experimental protocol was configured for testing. According to the relevant provisions of API SPE 10A [25], the thickening time of the cement slurry was defined as the duration from the initiation of the program to the point when the slurry consistency reached 100 Bearden units (Bc).

For compressive strength evaluation, the cement slurry was cast into a cubic mold (50.8 mm × 50.8 mm × 50.8 mm). After curing in a thermostatic water bath for a predetermined period, the specimen was demolded. Compressive strength was measured using a UTM5105X electronic universal testing machine (Shenzhen Sansi Zongheng Technology Co., Ltd., Shenzhen, China). The loading rate was controlled at 1.2 kN/s, with the specimen loaded uniformly until failure [23].

#### 2.2.5. Molecular Dynamics Simulation

The Materials Studio (MS) software of version 2020 was utilized to conduct full-atom molecular dynamics simulations of copolymer fluid loss agents. The Amorphous Cell module was employed to sequentially establish a segment model of the 5-membered copolymer SH5L, with a segment length set to 30 monomers. The model included 2000 water molecules and a specific amount of 36 Na^+^, 20 Ca^2+^, and 73 Cl^−^ ions, forming a full-atom solution model with periodic boundary conditions. The geometric structure of the molecular model was optimized at each step of the model construction process using the Forcite module under the COMPASS force field. Additionally, the simulations calculated the chain rotation radius (Rg) and radial distribution function (RDF). The NPT ensemble was used during the calculations to simulate the application environment of the copolymers. The last 80 ps are used for calculating the conformation of polymerization while reaching the system equilibrium.

## 3. Results

### 3.1. Structural Characterization of Copolymer Retarder

**Figure 2:**
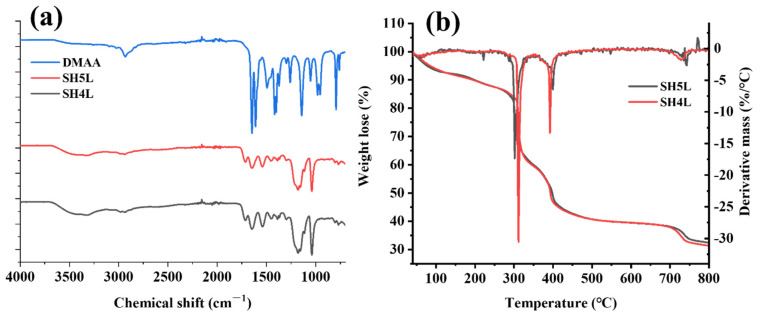
The FTIR of DMAA, SH4L and the SH5L (**a**); the TG and DTG of SH4L and SH5L (**b**).

To verify that the synthesized copolymer retarder is composed of the monomers used, the functional group composition was analyzed using infrared spectroscopy, and the thermal weight loss was measured using a thermogravimetric analyzer. The experimental results are shown in Figure 2.

The infrared spectra of retarders SH5L and SH4L are presented in Figure 2a. An absorption band observed at 3500 cm^−1^ is attributed to the stretching vibration of the -NH group in 2-acrylamido-2-methylpropanesulfonic acid (AMPS) and dimethylacrylamide (DMAA). The bending vibration of the -CH_2_- group and the stretching vibration of the -CH_3_ group are located at 2979 cm^−1^ and 2940 cm^−1^, respectively [26]. A distinct stretching vibration band at 1650 cm^−1^ corresponds to the C=O group in the amide bond of DMAA. For the carboxylate monomer, the symmetric stretching vibration of the -COO^−^ group is observed at 1550 cm^−1^, while its antisymmetric stretching vibration appears at 1440 cm^−1^. The stretching vibration of the C-N bond within the amide group is assigned to a band at 1213 cm^−1^. Additionally, the asymmetric stretching vibration of the -SO_3_^2−^ group in AMPS is displayed at 1044 cm^−1^ [27]. Notably, the characteristic peak of the cationic N^+^ in methacrylamidopropyltrimethylammonium chloride (MAPTAC) is identified at 910 cm^−1^, and an absorption band for the S-O bond is observed at 628 cm^−1^. These spectral features collectively indicate that the developed retarder SH5L is primarily composed of monomers including AMPS, itaconic acid (IA), DMAA, N-vinylpyrrolidone (NVP), and MAPTAC, with the characteristic functional groups of these monomers being clearly present in the spectrum.

The molecular weight and distribution of SH4L and SH5L were tested using GPC. The test results showed that the Mw of SH5L was 18,435.076, Mz was 18,656.836, and PDI was 1.0118. For SH4L, the Mw was 63,970.87, Mz was 93,930.374, and PDI was 1.8408. The lower molecular weight of SH5L results in a more stable state at high temperatures. Figure 2b presents the thermogravimetric (TG) and derivative thermogravimetric (DTG) curves of retarders SH4L and SH5L. For SH4L, a notable change in mass was observed at 304 °C, accompanied by a sharp increase in the absolute value of the TG curve’s slope. Concurrently, the DTG curve exhibited a pronounced decline, attaining the maximum decomposition rate at 311 °C. Subsequently, the second maximum decomposition rate was reached at 392 °C, indicating that SH4L remained relatively stable at 304 °C, while significant chain breakage occurred at 392 °C. In contrast, SH5L demonstrated its initial mass loss at 286 °C, with the DTG curve showing a sharp decrease at 301 °C. But the second and second maximum decomposition rates occur at 399 °C. The polymer chains of SH5L have a main titled stability below 286 °C, while the stability of the main chain structure is better than that of SH4L [28]. The weight loss rate and total weight loss rate of SH5L in the low-temperature region are lower than those of SH4L; DMAA molecules contain highly polar amide groups (-CON (CH3)_2_), which can form hydrogen bonds with active groups on the SH4L molecular chain, thereby enhancing initial thermal stability. SH4L exhibits sharp and steep DTG peaks at 200–400 °C, while the DTG peak of SH5L significantly widens and decreases, resulting in a smoother weight loss process. The amide group of DMAA may preferentially undergo side group decomposition at 200–400 °C, consuming some heat and producing gaseous products, which inhibits the severe decomposition of the SH5L main chain.

### 3.2. Effect of DMAA on Retarder Thickening Performance

To clarify the effect of DMAA introduction on the high-temperature thickening performance of retarders, a four-component polycarboxylate retarder was synthesized with the incorporation of DMAA at varying ratios of 0–0.8 (when the total amount of monomers is 10), and quaternary retarder SH4L (the DMAA is 0) was used as a control. The thickening time of cement slurry under different conditions was systematically tested under different retarders, which is synthesized with different DMAA ratios. The cement slurry composition is 600 g cement + x g retarder + (315 − x) g water + 18 g fluid loss agent + 6 g stabilizer + 210 g quartz sand +1.8 g dispersant. The experimental results are shown in Table 2.

At 180 °C, the thickening time of cement slurry without added retarder is 64 min, and on-site construction usually requires at least 3 h of safe time. Cement slurry without added retarder cannot meet the requirements of safe operation, the SH5L retarder containing 0.5/10 mol DMAA (at a dosage of 2.7 wt%) exhibited a thickening time of 268 min, representing a 20.15% increase compared to SH4L without DMAA. When the dosage of SH5L was increased to 3%, its thickening time (347 min) approached that of SH4L at 3.5% dosage, indicating that the incorporation of DMAA effectively enhanced its high-temperature performance, significantly prolonging the cement slurry thickening time even at a low dosage. Furthermore, as the dosage of SH5L increased from 2.7% to 3.0%, the thickening time extended to 347 min, demonstrating the advantage of “low dosage, prolonged thickening” a feature that reduces cementing costs and mitigates the adverse effects of excessive retarders on cement stone strength.

The amide group (-CON(CH_3_)_2_) in the DMAA molecule, compared to the carboxyl group (-COOH) in traditional polycarboxylate retarders, possesses superior thermal stability and hydrogen bond donor capability. At 180 °C, carboxyl groups are prone to dehydration or oxidative cleavage, leading to a reduction in the number of effective functional groups capable of chelating Ca^2+^ [20,21]. In contrast, the amide group resists molecular chain scission induced by high-temperature thermal motion through the conjugation effect of the C-N bond and the lone pair electrons of the N atom. Simultaneously, its N-H bonds can form a strong hydrogen bond network with oxygen atoms in cement hydration products, physically blocking the contact between water molecules and cement minerals. Moreover, the hydrolysis of amide groups in alkaline environments at high temperatures to form carboxyl groups can further enhance the chelating effect on cement particles, thereby delaying the hydration reaction rate [27].

### 3.3. Effect of DMAA on the Mechanical Properties of Cement Samples

**Figure 3:**
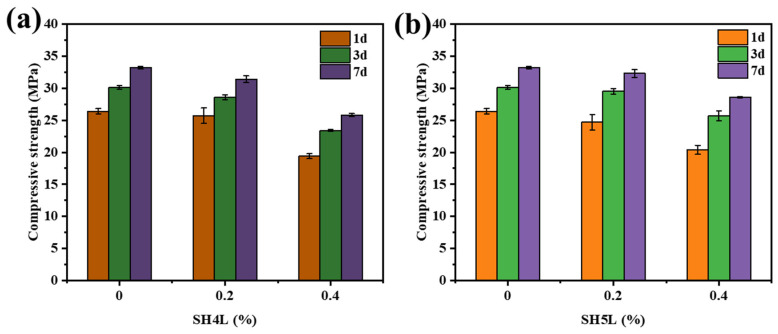
The effect of quaternary copolymer retarder on the strength of cement samples (**a**); the retarder introducing DMAA retarder on the strength of cement samples (**b**).

To elucidate the impact of DMAA incorporation on cement stone strength development, systematic tests were conducted on cement stones prepared with retarders containing DMAA (SH5L) and those without DMAA (SH4L). Compressive strengths were measured at key ages (1 d, 3 d, and 7 d) under 60 °C; the composition of the cement slurry is 600 g cement + y g retarder + (264 − y) g water; the results are shown in Figure 3.

**Figure 4:**
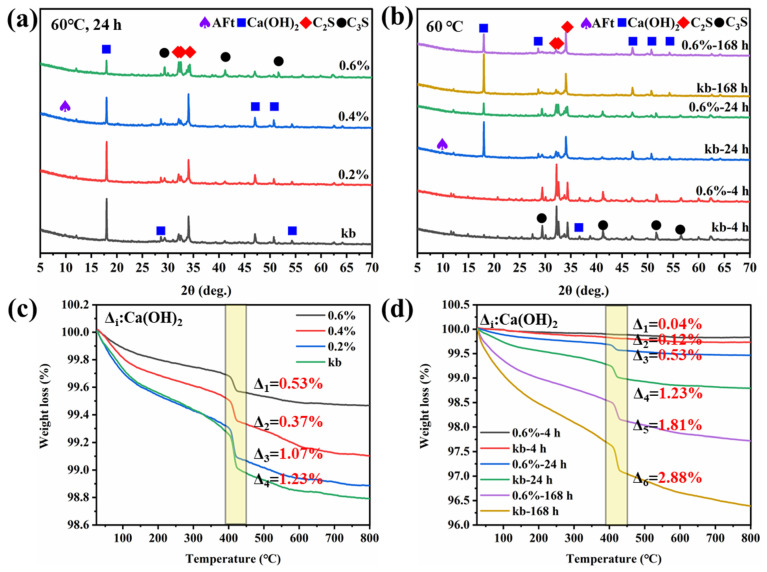
The XRD of different dosages of SH5L on the cement samples (**a**); the XRD of different times of SH5L on the cement samples (**b**); and the TGA of different dosages of SH5L on the cement samples (**c**); the TGA of different times of SH5L on the cement samples (**d**).

As shown in Figure 3, the compressive strength of SH5L containing DMAA is slightly lower than that of SH4L at 1d. This is consistent with the effect of retarders [29]. Adsorption and complexation of sulfonic acid groups and carboxyl groups in SH5L on cement particles delay the initial hydration and slow down the formation of early hydration products (such as ettringite AFt, C-S-H gel, as show in Figure 4) [13,15]. At 3–7 days, although the strength of SH5L and SH4L is lower when the dosage is the same, it is very close and consistent with the strength development of blank cement stone. It is worth noting that SH5L has a better retarding effect; therefore, at the same thickening time, the content of SH5L is relatively lower and the strength development is relatively faster. DMAA’s amide group (-CON (CH_3_)_2_) introduces the “intelligent retarding” mechanism: at low temperatures, it forms weak hydrogen bonds with C-S-H gel precursors, slightly delaying nucleation; at high temperatures, amide-based water can release carboxyl groups under alkaline conditions, further promoting the adsorption and complexation of cement slurry particles, and improving the high-temperature retarding ability.

### 3.4. Phase Composition of Cement Samples

The phase composition changes in the cement slurry with SH5L were analyzed using X-ray diffraction (XRD) and thermogravimetric analysis (TG); the experimental results are shown in Figure 4.

Figure 4a reveals that the characteristic peaks of calcium hydroxide (Ca(OH)_2_) and hydrated calcium silicate in cement samples become less prominent after adding SH5L, indicating reduced quantities of Ca(OH)_2_ and ettringite crystals [30,31]. The incorporation of SH5L inhibits the growth of Ca(OH)_2_ crystals, with this inhibition becoming more pronounced as the dosage of SH5L increases. Figure 4b shows that the characteristic peaks of Ca(OH)_2_ in cement paste with SH5L are lower than those in plain cement paste at 4 h and 24 h, suggesting that SH5L significantly retards hydration in the early stages. The presence of Ca(OH)_2_ in the cement becomes more evident at 168 h, indicating that SH5L primarily inhibits early hydration and has no significant effect in later stages. The carboxyl groups in the polymer chelate calcium ions early in cement hydration, forming stable chelates or insoluble salts, thereby delaying the growth of crystals such as calcium hydroxide and hindering the hydration process. However, in later hydration stages, cement particles fully contact each other, weakening the coupling effect of polymers on ions and allowing cement particles to form a dense structure [32,33].

The weight loss of cement at 390–450 °C is attributed to the decomposition of calcium hydroxide crystals, which reflects the relative content of calcium hydroxide [34]. Thermo gravimetric (TG) tests shown in Figure 4c,d also confirm that the addition of SH5L mainly affects the growth of Ca(OH)_2_ crystals in early cement hydration. The Ca(OH)_2_ content in cement paste decreases at 24 h as the dosage of SH5L increases, while the differences in crystal content are minimal in later hydration stages. The characteristic effects of SH5L on the phase composition of cement paste result in an extended thickening time of the cement paste upon adding SH5L, without significantly compromising its long-term mechanical properties.

### 3.5. Microscopic Morphology of Cement Samples

**Figure 5:**
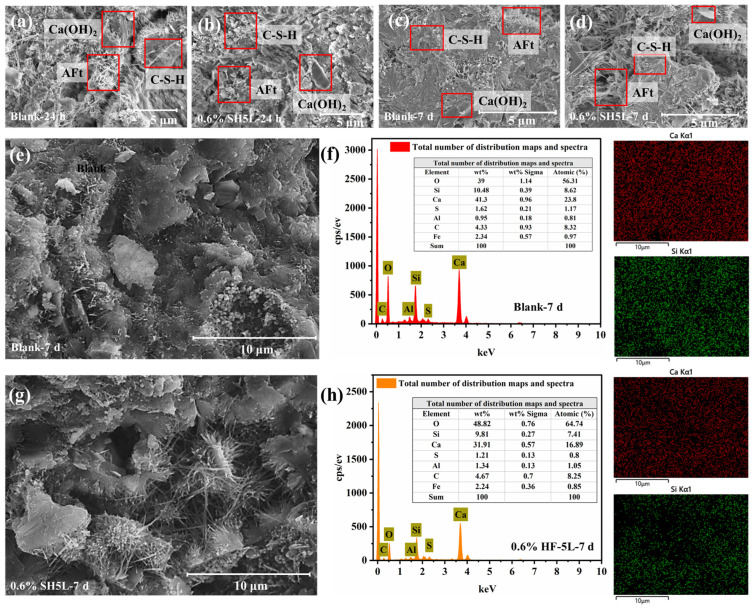
(**a**–**d**) SEM of cement samples with different dosages of retarder SH5L with different curing time; (**e**,**f**) the element scanning area and distribution of blank cement stone after 7 days of curing; (**g**,**h**) the element scanning area and distribution of cement stone with 0.6% SH5L after 7 days of curing.

To further investigate the effect of SH5L on the structure of cement slurry, scanning electron microscopy (SEM) was used to analyze the microstructure of cement samples with different retarders. Energy-dispersive X-ray spectroscopy (EDS) tests were also performed on cement samples cured for 7 days. The test results are shown in Figure 5.

Comparing Figure 5a,b, the blank cement samples at 24 h exhibited fewer pores, clear acicular ettringite crystals, and interwoven platelet calcium hydroxide crystals, indicating good cement hydration. The cement samples with SH5L showed a relatively loose structure, with fewer acicular crystals and platelet calcium hydroxide, and mostly amorphous substances, indicating that the copolymer retarder SH5L in cement slurry further inhibits the hydration process of cement slurry by suppressing the growth of calcium hydroxide and ettringite [30,35]. Comparing Figure 5c,d, both samples had similar pore contents after 7 days of curing. However, the plain cement paste had a denser structure, while the retarded cement paste contained abundant acicular ettringite and bulky calcium hydroxide. This indicates that early cement hydration was inhibited by the retarder, but with extended hydration, the hydration products developed well. Figure 5f,h show the EDS scan results of Figure 5e in the area after curing with blank cement stone for 7 days and Figure 5g in the area after adding 0.6% SH5L and curing for 7 days. It was revealed that after 7 days of curing, the cement sample with SH5L had higher oxygen content and a calcium-to-silicon ratio (CaO/SiO_2_ mass ratio) of approximately 3.25. The blank cement samples had the highest calcium content and a Ca/Si ratio of about 3.94, indicating more Ca(OH)_2_, which enhances cement paste strength. The cement samples with the retarder had more carbon due to the addition of the polymer, slightly inhibiting the formation of hydrated calcium silicate. Carboxylate-containing polymer retarders can chelate with calcium ions or form insoluble salts during cement hydration, slowing early hydration but allowing the formation of dense structures with extended hydration. This mechanism ensures that the retarder does not significantly reduce the mechanical properties of the cement [36,37].

### 3.6. Microscopic Pore Structure of Cement Samples

**Figure 6:**
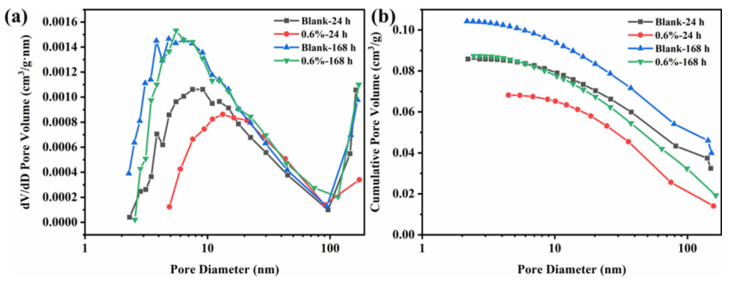
Pore volume change rate (**a**) and cumulative pore volume (**b**) of cement samples.

To further study the influence of polymer retarder SH5L on the hydration process and pore structure of cement at the microscopic level, nitrogen isothermal adsorption and desorption testing were conducted on blank cement and cement samples with 0.6% SH5L. The experimental results are shown in Figure 6.

Figure 6 shows that the number of gel micropores in blank cement and cement with 0.6% SH5L increases as the curing time of cement increases from 24 h to 168 h, indicating the growth of calcium silicate hydrate [38]. At 24 h, the cumulative pore surface area of blank cement was 10.9558 m^2^/g, and the cumulative pore surface area of cement with 0.6% SH5L was 8.3968 m^2^/g. The peak pore size distribution of blank cement is at 9.12 nm, while the peak pore size distribution of cement with 0.6% SH5L appears at 13.29 nm. The addition of retarders has to some extent inhibited the development of cement hydration calcium silicate. As time goes on, the cumulative pore surface area of blank cement at 168 h is 15.3402 m^2^/g, and the cumulative pore surface area of cement with 0.6% SH5L is 13.87992 m^2^/g. The peak pore size distribution of blank cement is 4.82 nm, while the peak pore size distribution of cement with 0.6% SH5L appears at 5.52 nm. As the hydration time prolongs, the degree of inhibition of cement hydration by the retarder weakens, and the C-S-H inside the cement can develop well [39]. A small amount of SH5L inhibits the development of cement hydration calcium silicate to some extent, but has no significant adverse effect on the strength development of cement in the later stage.

## 4. Discussion

### 4.1. Analysis of Conformation of SH5L

**Figure 7:**
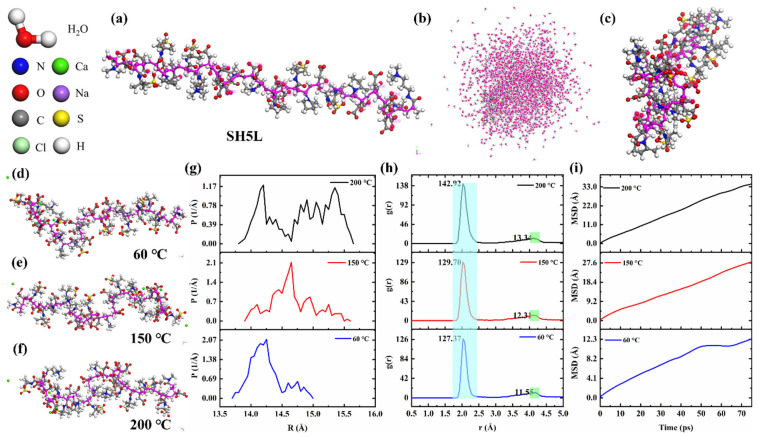
(**a**–**c**) The structure and dispersion state of copolymer retarder SH5L in Ca^2+^ solution are displayed; (**d**–**f**) dispersion state of copolymer retarder SH5L at different temperatures; (**g**) chain turning radius of SH5L at different temperatures; (**h**) radial basis functions of SH5L at different temperatures; (**i**) diffusion coefficient of SH5L at different temperatures.

Polymer retarder mainly affects the hydration of cement through electrostatic adsorption between cement particles and hydration products, as well as the chelating effect of polymer functional groups on Ca^2+^ [40]. The retarding effect of polymers on cement varies at different temperatures, mainly due to changes in molecular conformation. To further investigate the retarding mechanism of polymer retarders on cement, molecular dynamics simulations were used to study the conformational changes in polymers in cement slurry under different environmental conditions. The experimental results are shown in Figure 7.

Figure 7a shows the polymer retarder SH5L with 30 units. After sufficient annealing and energy optimization in a simulated cement slurry solution environment, the polymer conformation is shown in Figure 7c. It can be observed that in cement slurry, polymer chains exist in a curled-up form. To investigate the ion interaction and conformational changes in polymer retarder SH5L with cement slurry under different temperature conditions, kinetic simulations were conducted at 60 °C, 150 °C, and 200 °C. The experimental results are shown in Figure 7d–g and illustrate the chain rotation radius of the polymer in solution. It can be seen that as the temperature increases, the chain rotation radius of the polymer in the solution tends to increase, and the polymer chain transitions from a curled to a stretched conformation. Figure 7h shows the radial distribution function (RDF) of polymer carboxyl oxygen atoms and Ca^2+^ in the solution. When the distance between the oxygen-containing anionic group and the cation is less than 2.5 Å, it indicates the formation of an ionic bond between the two [41]. The results show that as the temperature increases, the interaction between carboxyl groups and Ca^2+^ weakens, leading to a macroscopic reduction in the retarding effect, explaining why SH5L exhibits better retarding effect at high temperatures [38].

### 4.2. Thickening Regulation Mechanism of SH5L

The sulfonic acid and carboxyl functional groups in SH5L induce strong adsorption onto cement particles, creating a protective gel layer that slows ion diffusion and delays the formation of AFt and C-S-H networks. The polymer chains transition from coiled to extended conformations at elevated temperatures, reducing intermolecular interactions and weakening Ca^2+^-carboxylate binding affinity, which paradoxically maintains effective retardation by balancing adsorption efficiency and chain mobility. The introduction of DMAA into the copolymer retarder SH5L significantly enhances its high-temperature retarding performance through a synergistic mechanism involving adsorption–complexation interactions and intelligent molecular dynamics. The amide group (-CON(CH_3_)_2_) in DMAA forms weak hydrogen bonds with C-S-H gel precursors at low temperatures, slightly delaying nucleation by stabilizing the amorphous phase of hydrated calcium silicate. As temperature rises, the amide–water interaction weakens, releasing carboxyl groups under alkaline conditions that amplify adsorption onto cement particles and promote chelation with Ca^2+^ ions, further inhibiting early-stage hydration. This dual action results in prolonged induction periods and extended hydration times under high-temperature conditions. Despite transient inhibition of early C-S-H development, prolonged hydration ultimately allows dense microstructural formation, with minimal impact on late-stage compressive strength due to controlled pore refinement and optimized hydration pathways. This intelligent retarding mechanism, combining dynamic adsorption modulation and temperature-adaptive molecular architecture, positions SH5L as a high-performance additive for deep-well cementing applications.

## 5. Conclusions

(1) The five-component polymeric retarder SH5L, prepared by incorporating the functional monomer DMAA, exhibits superior structural stability at high temperatures compared to the quaternary copolymeric retarder SH4L.

(2) At 180 °C, the cement slurry containing 3% SH5L demonstrated a thickening time exceeding 300 min, with the thickening curve remaining stable and exhibiting a sharp increase, which approached the thickening time of SH4L at a 3.4% dosage. Moreover, SH5L displayed better retardation performance at lower dosages. SH5L has no significant adverse effect on the mechanical properties of cement slurry at the same dosage, and the strength development in the later stage is close to that of blank cement slurry.

(3) The incorporation of SH5L inhibits the growth of Ca(OH)_2_ crystals, with this inhibition becoming more pronounced as the dosage of SH5L increases. SH5L primarily inhibits early hydration and has no significant effect in later stages. The carboxyl groups in the polymer chelate calcium ions early in cement hydration, forming stable chelates or insoluble salts, thereby delaying the growth of crystals such as calcium hydroxide and hindering the hydration process. The coupling of SH5L and Ca^2+^ retards the hydration and crystallization process of cement slurry. The combination of rigid polycyclic structures and cationic monomers weakens the chelation between anionic groups and Ca^2+^, inhibiting the curling of the polymer in ionic solutions. As temperature increases, the polymer chains stretch, enhancing their ability to bind with Ca^2+^ and improving their high-temperature retardation performance. At elevated temperatures, the amide group in DMAA hydrolyzes to generate carboxyl groups, further enhancing its retardation capacity under high-temperature conditions.

(4) This study provides technical support for the design of high-temperature retarders, improving the temperature resistance and high-temperature retardation capacity of cement slurries. However, this polymeric retarder is highly sensitive to dosage at high temperatures, necessitating further research to develop retarders with excellent high-temperature stability and reduced sensitivity under extreme conditions.

## Figures and Tables

**Table 1:** Composition of G-class cement.

Component	CaO	SiO_2_	Al_2_O_3_	Fe_2_O_3_	MgO	SO_3_	K_2_O	Others
Percentage (%)	63.05	20.63	3.54	5.87	1.62	3.771	0.534	0.985

**Table 2:** Thickening time of retarders with different DMAA ratios.

Retarder Type	DMAA Ratio in Retarders (Controlled 10)	Retarder’s Dosage (%)	Temperature (°C)	Thickening Time (min)
Blank	-	0	180	64
SH4L	0	2.7	180	214
SH4L	0	3.4	180	356
SH5L	0.2	2.7	180	253
SH5L	0.5	2.7	180	268
SH5L	0.5	3.0	180	347
SH5L	0.8	2.7	180	264

## Data Availability

The original contributions presented in this study are included in the article. Further inquiries can be directed to the corresponding author.

## Abbreviations

The following abbreviations are used in this manuscript:

SH4L	High temperature retarder formed by copolymerization of four monomers
SH5L	High temperature retarder formed by copolymerization of five monomers
DMAA	N,N-dimethylacrylamide
C_3_A	Tricalcium aluminate
AMPS	2-acrylamido-2-methylApropanesulfonic acid
NVP	N-vinylpyrrolidone
MAPTAC	Methacryloyl propyl trimethylammonium chloride
APS	Ammonium persulfate
NaOH	Sodium hydroxide
IA	Itaconic acid
FTIR	Fourier transform infrared
TGA	Thermogravimetric analysis
XRD	X-ray diffraction
SEM	Scanning electron microscopy
MS	Materials Studio
RDF	Radial distribution function
Rg	Chain rotation radius
DTG	Derivative Thermogravimetry
AFt	Alumino-Ferrite tri
C-S-H	Calcium Silicate Hydrate
Ca(OH)_2_	Calcium hydroxide
EDS	Energy-dispersive X-ray spectroscopy
